# Contribution of type 2 diabetes associated loci in the Arabic population from Tunisia: a case-control study

**DOI:** 10.1186/1471-2350-10-33

**Published:** 2009-04-15

**Authors:** Intissar Ezzidi, Nabil Mtiraoui, Stéphane Cauchi, Emmanuel Vaillant, Aurélie Dechaume, Molka Chaieb, Maha Kacem, Wassim Y Almawi, Philippe Froguel, Touhami Mahjoub, Martine Vaxillaire

**Affiliations:** 1 Research Unit of Biology and Genetics of Hematological and Auto-immune diseases, Faculty of Pharmacy of Monastir, University of Monastir, Monastir, Tunisia; 2 Higher Institute of Biotechnology of Monastir, University of Monastir, Monastir, Tunisia; 3 Genomics and Molecular Physiology of Metabolic Diseases, CNRS, 8090-Lille Institute of Biology, Pasteur Institute, Lille, France; 4 Endocrinology and Diabetic Service, CHU Farhat Hached of Sousse, Tunisia; 5 Nephrology and Internal Medicine Service, CHU Fatouma Bourguiba, Monastir, Tunisia; 6 Department of Medical Biochemistry, Arabian Gulf University, Manama, Bahrain; 7 Genomic Medicine, Hammersmith Hospital, Imperial College, London, England, UK

## Abstract

**Background:**

Candidate gene and genome-wide association studies have both reproducibly identified several common Single Nucleotide Polymorphisms (SNPs) that confer type 2 diabetes (T2D) risk in European populations. Our aim was to evaluate the contribution to T2D of five of these established T2D-associated loci in the Arabic population from Tunisia.

**Methods:**

A case-control design comprising 884 type 2 diabetic patients and 513 control subjects living in the East-Center of Tunisia was used to analyze the contribution to T2D of the following SNPs: E23K in *KCNJ11/Kir6.2*, K121Q in *ENPP1*, the -30G/A variant in the pancreatic β-cell specific promoter of Glucokinase, rs7903146 in *TCF7L2 *encoding transcription factor 7-like2, and rs7923837 in *HHEX *encoding the homeobox, hematopoietically expressed transcription factor.

**Results:**

*TCF7L2*-rs7903146 T allele increased susceptibility to T2D (OR = 1.25 [1.06–1.47], *P *= 0.006) in our study population. This risk was 56% higher among subjects carrying the TT genotype in comparison to those carrying the CC genotype (OR = 1.56 [1.13–2.16], *P *= 0.002). No allelic or genotypic association with T2D was detected for the other studied polymorphisms.

**Conclusion:**

In the Tunisian population, *TCF7L2*-rs7903146 T allele confers an increased risk of developing T2D as previously reported in the European population and many other ethnic groups. In contrast, none of the other tested SNPs that influence T2D risk in the European population was associated with T2D in the Tunisian Arabic population. An insufficient power to detect minor allelic contributions or genetic heterogeneity of T2D between different ethnic groups can explain these findings.

## Background

T2D is a complex metabolic disorder which is caused by both decreased insulin sensitivity, and impaired insulin secretion due to pancreatic β-cell defects [1]. T2D is thought to result from the effects of environmental and lifestyle risk factors together with at-risk genetic variants in predisposed individuals [2].

T2D is a global major health problem showing worldwide increasing prevalence [3]. The Arabic population is however particularly targeted by T2D [4,5]. In the Tunisian population, the prevalence of T2D reaches 9% of adults [6] that is much higher compared with European populations and may be due to the specificity of the Tunisian life style or to a specific genetic background.

From the previous familial linkage and candidate-gene studies, T2D-associated single nucleotide polymorphisms (SNPs) have been confirmed and widely replicated, but with modest effects on disease risk [7,8]. These variants include the E23K variation in *KCNJ11*, encoding the Kir6.2 subunit of the K^+^-ATP channel [9], the Pro12Ala variant in *PPARG *[10], the -30G/A polymorphism in the β-cell specific promoter of glucokinase *(GCK) *[8,11], and the K121Q variant of *ENPP1 *encoding ectonucleotide pyrophosphatase phosphodiesterase, the inhibitor of insulin receptor [12].

The SNP with the largest risk effect is the intronic variant, rs7903146, in the *TCF7L2 *locus [13,14]. This association was consistently replicated in populations of various ethnic origins, among which Morrocans [15].

Recently, genome-wide association (GWA) studies revealed novel SNPs that increased T2D risk in different European populations [14,16-18]. The French GWA study detected unexpected association to T2D for non-coding SNPs at the *HHEX *locus (homeobox, hematopoietically expressed) [14], which were also shown to contribute to an increased risk of T2D in British [18], Japanese [19] and other Asiatic [20] populations.

In this study, we analyzed five polymorphisms in the following genes, rs7903146 of *TCF7L2*, rs7923837 of *HHEX*, rs1788994 of *GCK*, rs5219 of *KCNJ11/Kir6.2 *and rs1044498 of *ENPP1 *using a case-control design in 1,397 individuals (884 unrelated T2D patients and 513 normoglycemic controls) to assess their association with T2D risk in the Tunisian population. To our knowledge, four of them have not been previously tested in this Arabic population, and we aim to evaluate whether these common variants reported to be at-risk for T2D in European populations may also contribute to T2D risk and aetiology in the Tunisian population living in the East-Center part of the country.

## Methods

### Study population

The T2D group includes 884 unrelated Tunisian diabetic subjects (406 males, 478 females). The affected individuals were recruited in 2003–2006 in collaboration with the Endocrinology-Diabetology departments of Farhat Hached Hospital (Sousse, Tunisia) and Fattouma Bourguiba University Hospital (Monastir, Tunisia). T2D was defined according to 1997 American Diabetes Association. Inclusion criteria: fasting plasma glucose ≥ 7.0 mmol/l and/or treatment for diabetes included diet and/or oral antidiabetic drugs and/or insulin to achieve glycemic control. All subjects who required insulin had been treated with oral drugs for at least 2 years.

The diabetic cases included in the study are representative of the diabetic population examined in two hospital clinics in the center of Tunisia (Sousse and Monastir, Tunisia) within a time period of 4 years; no clinical criteria of exclusion have been held (except patients diagnosed with type 1 diabetes, and patients with type 2 diabetes diagnosed at age ≤ 40 years).

Individual and clinical characteristics were recorded for all subjects, including age at examination, gender, age at diagnosis, duration of diabetes, first-degree family history of diabetes, treatment for diabetes including date of initiation and/or discontinuation of oral agents or insulin. When available, the following details were obtained from the clinic records: dyslipidaemia, history of chronic complications of diabetes, history of hypertension, ischaemic heart disease and other medical illness.

All T2D patients were compared to a group of 513 normoglycaemic subjects (fasting glycaemia < 6.1 mmol/l, age at examination > 45 years, BMI < 30 kg/m^2^) from blood donors recruited in the transfusion centres of Monastir and Sousse (Center of Tunisia). None was first degree relative of other subjects in the case or control groups; they were not known to have diabetes although occult disease was not excluded.

Written informed consent was obtained from all subjects and DNA was extracted using the standard phenol-chloroform procedure. The study protocol was approved by the University of Monastir (Tunisia).

### SNP genotyping

SNP genotyping of rs7903146 in *TCF7L2*, rs7923837 in *HHEX *and rs1799884 in *GCK *promoter were performed using allelic discrimination TaqMan SNP Genotyping Assays (Applied Biosystems, Foster City, California. USA). The PCR primers and TaqMan probes were designed by Primer Express and optimized according to the manufacturer's protocol. We obtained a 95% genotyping success rate (except for *HHEX *rs7923837 which gave a 90% genotyping rate). A random of 10% sample set was re-tested with the same method to confirm genotype accuracy. No difference of genotypes was found between the duplicate samples.

For genotyping of E23K (rs5219) in *KCNJ11/Kir6.2 *and K121Q (rs1044498) in *ENPP1*, we used the FRET technology using the Light Cycler TM assay (Roche Diagnostics, Basel, Switzerland). For both SNPs, the genotyping success rate was 91%. In order to assess genotyping accuracy for these two SNPs, 20 random samples were tested by direct sequencing, which provided a 100% concordance rate.

### Statistical analysis

Allele frequencies were calculated by the genotype-counting method, and each polymorphism was tested for Hardy-Weinberg equilibrium using Chi square goodness-of-fit test using HPlus 2.5 software (see Additional file 1). Comparison of allele frequencies and genotype distributions between all T2D and control groups were done using the Pearson's Chi square test.

Genotypic associations for additive, dominant and recessive models were tested by calculating a logistic regression (adjustments) statistic and corresponding *P *value using the program SNPstats . The results are expressed as *P *value (two-tailed), odds ratio (OR) and 95% confidence intervals (CI). The minimum detectable effect size with a statistical power of 80% was assessed [21] using Quanto software v.1.2.3 .

Student's t-test, used to determine differences in means of continuous variables in the normoglycemic control subjects, was performed using the SPSS statistical analysis software v.16.0 (SPSS, Chicago, Illinois, USA). Statistical significance was set at a *P*-value < 0.05.

## Results

The clinical characteristics of the T2D patients and control subjects are given in Table 1. Among T2D subjects, 30.09% (n = 266) are obese (BMI ≥ 30 kg/m^2^) versus 69.91% (n = 618) non-obese (BMI < 30 kg/m^2^). No significant differences in clinical features were noted when all T2D patients were compared to the non-obese T2D group (data not shown).

**Table 1:** Characteristics of the T2D patients and control subjects from the Tunisian population

Characteristics	**Controls (n = 513)**	**T2D (n = 884)**
Gender (Male/Female)	258/255	406/478
Age at examination (years)	60 ± 8.69	59.42 ± 11.09
Mean BMI (kg/m2)	24.83 ± 2.73	27.82 ± 5.30
Systolic blood pressure (mmHg)	122.12 ± 14.33	139.80 ± 28.13
Diastolic blood pressure (mmHg)	78.18 ± 10.55	80.92 ± 12.73
Fasting glucose (mmol/l)	5.05 ± 0.64	12.67 ± 5.30
HbA1c (%)	4.47 ± 1.23	9.49 ± 3.89
Total cholesterol (mmol/l)	4.64 ± 1.28	5.26 ± 1.42
Triglycerides (mmol/l)	1.18 ± 0.60	1.77 ± 1.31
HDL-cholesterol (mmol/l)	1.27 ± 0.39	1.07 ± 0.38
LDL-cholesterol (mmol/l)	2.59 ± 1.60	3.77 ± 1.37

n: number of total subjects

Data are expressed as means ± SD.

Most of the diabetic patients included in the study were recruited immediately after their admission to the Endocrinology Department, explaining a poor control of glycemic levels (mean fasting glucose: 12.67 mmol/l; mean HbA1c: 9.49%) in these subjects.

The distribution of allelic and genotypic frequencies of the five SNPs was compared between the two study groups (884 T2D cases and 513 normoglycemic controls) (Table 2). The genotype distributions of all SNPs obeyed Hardy Weinberg equilibrium in the control group (Supplementary Table).

**Table 2:** T2D association for candidate SNPs in the Tunisian study sample of 1,397 individuals

**Gene SNP**	**Genotype**	**Control/T2D**	**Additive model**^1^ **OR (95% CI)**	***P***^†^	**Dominant model**^2^ **OR (95% CI)**	***P***^‡^	**Recessive model**^3^ **OR (95% CI)**	P*
*TCF7L2 *rs7903146	n	511/863						
	CC (1/1)	181/250						
	CT (1/2)	235/396	1.24 (0.95–1.61)	0.135				
	TT (2/2)	95/217	1.56 (1.13–2.16)	0.002	1.33 (1.04–1.70)	0.023	1.38 (1.04–1.83)	0.025
	MAF (T)	0.42/0.48	1.25 (1.06–1.47)	0.006^#^				

*KCNJ11 *rs5219	n	503/805						
	CC (1/1)	250/371						
	CT (1/2)	213/352	1.03 (0.80–1.31)	0.399				
	TT (2/2)	40/82	1.23 (0.80–1.90)	0.148	1.06 (0.84–1.34)	0.630	1.22 (0.80–1.85)	0.360
	MAF (T)	0.29/0.32	1.07 (0.90–1.29)	0.440^#^				

*GCK*****rs1799884	n	505/865						
	GG (1/1)	324/552						
	GA (1/2)	157/272	1.01 (0.79–1.30)	0.939				
	AA (2/2)	24/41	1.20 (0.69–2.08)	0.902	1.03 (0.81–1.32)	0.780	1.20 (0.70–2.05)	0.510
	MAF (A)	0.20/0.20	1.04 (0.86–1.28)	0.640^#^				

*HHEX*****rs7923837	n	504/795						
	GG (1/1)	271/448						
	GA (1/2)	200/292	0.88 (0.69–1.13)	0.661				
	AA (2/2)	33/55	0.97 (0.60–1.58)	0.934	0.89 (0.70–1.13)	0.340	1.03 (0.64–1.65)	0.910
	MAF (A)	0.26/0.25	0.93 (0.77–1.13)	0.470^#^				

*ENPP1 *rs1044498	n	499/809						
	AA (1/1)	228/402						
	AC (1/2)	205/311	0.86 (0.67–1.12)	0.243				
	CC (2/2)	66/96	0.78 (0.54–1.14)	0.326	0.84 (0.66–1.07)	0.160	0.84 (0.59–1.19)	0.320
	MAF (C)	0.33/0.31	0.88 (0.74–1.04)	0.140^#^				

n: number of total subjects, MAF: Minor allele frequency, ^†,‡^,*Genotype specific *P *values and OR are adjusted for age gender and BMI in each additive, dominant or recessive genetic model, respectively. ^#^Allele-specific *P *values and OR of the log-additive genetic model are adjusted for age, gender and BMI.

^1 ^Genetic additive model: 1/1 vs. 1/2 or 2/2 genotypes

^2 ^Genetic dominant model: 1/1 vs. 1/2 + 2/2 genotypes

^3 ^Genetic recessive model: 1/1 + 1/1 vs. 2/2 genotypes

The T allele of *TCF7L2 *was significantly associated with increased risk of T2D and the OR adjusted for age, gender and BMI was 1.25, 95% IC [1.06–1.47], *P *= 0.006 (Table 2). The association was observed in all genotypic models but the highest risk was observed under the additive model (OR = 1.56, 95% IC [1.13–2.16], *P *= 0.002) (Table 2).

As shown in Table 2, no other alleles of the remaining variants were associated with an increased risk of T2D in the Tunisian population.

We estimated the minimum effect sizes detectable with a statistical power of 80% under different genetic models, and according to the allelic frequencies of each SNP tested in our study, as indicated in Table 3. Except for *TCF7L2 *SNP that showed a significant effect on T2D risk with an additive OR value above the threshold estimated in our power calculation, the other four SNPs are known to have a lower allelic contribution that could not be easily detectable in this middle-sized cohort (Table 3).

**Table 3:** Minimum effect size detected with a statistical power of 80% in the study sample

**Gene name**	**SNP rs ID**	**Additive model** **OR (T2D)**	**Dominant model** **OR (T2D)**	**recessive model** **OR (T2D)**
*TCF7L2*	rs7903146	1.25	1.44	1.43
*KCNJ11*	rs5219	1.26	1.37	1.62
*GCK*	rs1799884	1.31	1.38	2.05
*HHEX*	rs7923837	1.30	1.40	1.80
*ENPP1*	rs1044498	1.27	1.37	1.64

The estimation of the risk effects (OR) with a statistical power of 80% was assessed using the Quanto software.

T2D: Type 2 diabetes

## Discussion

The T-allele of *TCF7L2*-rs7903146 is associated with an increased risk in T2D among the Tunisian population. Recent GWA studies in populations of European descent showed that *TCF7L2 *is the T2D gene having the largest risk effect to date [14,22], even if the causative variant(s) and etiological mechanism(s) are not yet completely characterized [23]. Our data are also in agreement with the numerous previous replications of the *TCF7L2*-associated SNPs mostly in white Europeans, but also in West Africans, Mexican and African Americans, Indians and Japanese populations [13,15,24]. The rs7903146 variant was also associated with an increased risk in T2D in Moroccan subjects, suggesting a similar effect in several North African populations [15]. However, a recent study of *TCF7L2 *variants in an Arab population of Saudi origin reported no association with T2D upon analysis of two SNPs (including rs7903146) [25].

With regard to the remaining four loci, no association with T2D was detected for *HHEX*, *GCK*, *KCNJ11 *and *ENPP1 *variants, whereas previous independent studies reported such associations with T2D risk in several European white populations [9,14,26,27]. However, a lack of association between SNPs at the *HHEX *locus and T2D was also reported in a Moroccan population [27].

Minor allele frequencies of the SNPs examined in this study compared to those reported from several other populations with different ethnic backgrounds appeared to be variable depending on ethnicity. For example, the frequency of the Q121 allele in our study population is higher (31%) than the one reported in Caucasians which ranged from 10 to 17% [12,28], but relatively lower than the one observed in another Tunisian cohort living in the north of the country (47%) [29], or in African Americans (78,5%) [30].

The extensively studied K121Q variant (rs1044498) in *ENPP1 *did not reveal evidence for association with T2D in our study from the Tunisian population. The contribution of this variant to T2D risk in the European white populations has been established in several [12,31] but not all [32,28] studies. These findings question the reproducibility of a real contribution to T2D risk and emphazise the more complex diabesity component.

A recent study in another Tunisian population reported that the *ENPP1*-K121Q variant may predispose to T2D (with an OR of 1.89, 95%CI [1.13–3.15], under a dominant model) [29], but the smaller sample size and ascertainment of the diabetic patients analyzed could explain this difference. In addition, it is noteworthy that in our study we investigated a multicentric sample of T2D patients, more representative of the Tunisian general population contrary to the study by Bouhaha et al. which investigated only a group of individuals from the north of Tunisia. In this part of country, people life style's is more westernized with reduced physical activity and excessive calorie intake in foods. Otherwise, these divergent results of association, whereas a similar frequency of the Q-121 allele was observed in both studies, could be explained by a modest contribution of the *ENPP1*-K121Q variant to the risk of T2D in Tunisians at the population level, and also in interaction with BMI and environmental factors in modulating T2D risk, as previously reported [33].

We have to note that the present study was underpowered to demonstrate an effect in T2D risk for *HHEX*, *GCK*, *KCNJ11 *or *ENPP1 *variants similar to those previously reported from the European diabetic cohorts [27,30,34]. Indeed, more modest genetic effects in a polygenic context will need analysis of a larger sample size to be able to definitely conclude for a lack of association due to genetic heterogeneity between specific ethnic backgrounds or to other confounding factors, or to an underpowered association study.

It was previously demonstrated that the genetic effects of variants associated with either insulin secretion (like for *GCK, KCNJ11 or TCF7L2*) or insulin action (for *ENPP1, ADIPOQ or PPARG*) may be modulated by the obesity status or adiposity [9,12,26,28,32,34]. Thus, the impact on T2D risk may be largely different in obese and non obese individuals depending on the gene variant, as this was exemplified in adequately designed studies [12,26,28,34].

Previous genetic studies undertaken in this case-control study from the Tunisian population have assessed the impact of a number of gene variants on T2D risk and vascular complications, such as *PPARG *[35], *eNOS *[36], *MTHFR *[37], *IL-10 *[38]. Except to the Pro12Ala variants of *PPARG *which were found to be associated only to a lower BMI among T2D patients, the other variants showed a contribution to T2D and in part to diabetic vascular complications.

## Conclusion

In conclusion, our data support an effect of the widely replicated *TCF7L2 *variant on T2D risk in the Arabic population from Tunisia, whereas the other variants tested were not found to play a major role in T2D. In comparison to the European and non-European populations, these findings can be explained by several factors, such as a minor contribution of the studied variants that is not detectable in this middle-sized cohort, the presence of Arabic-specific SNPs in some loci, or a genetic heterogeneity of T2D between different ethnic groups, which we already highlighted in a recent study of novel T2D-associated SNPs in several populations of different ethnic origins [27]. In this context, further GWA studies in Arabic populations from Maghreb are required to further define the genetic components of T2D in these populations.

## Abbreviations

BMI: Body Mass Index; CI: Confidence interval; K^+^-ATP channel: ATP-sensitive potassium channel; MAF: Minor Allele Frequency; OR: Odds ratio; PCR: Polymerase Chain Reaction; SNP: Single Nucleotide Polymorphism; T2D: Type 2 Diabetes.

## Competing interests

The authors declare that they have no competing interests.

## Authors' contributions

IE and NM participated in the design of the study, carried out the SNP genotyping and the analyses of the genotype data, and contributed to the statistical analyses and the drafting of the manuscript. SC contributed to the statistical analyses and participated in the writing of the manuscript. EV participated in the SNP genotyping and some of the genetic analyses. AD carried out some of the genotyping experiments. MC and MK coordinated the patients' recruitment. WYA, PF and TM contributed to the manuscript editing. MV contributed to the design and coordination of the study, to the genetic analyses and drafted the manuscript. All authors read and approved the final manuscript.

## Pre-publication history

The pre-publication history for this paper can be accessed here:



## Supplementary Material

Additional file 1 **Supplemental table**. Test of Hardy-Weinberg equilibrium for each SNP genotyped in the control and T2D study subjects. Click here for file
