# Supplementary material for: Contribution of type 2 diabetes associated loci in the Arabic population from Tunisia: a case-control study
Source: BMC Med Genet. 2009 Apr 15;10:33. doi: 10.1186/1471-2350-10-33 (PMC2678106; doi:10.1186/1471-2350-10-33)
Supplement: Additional file 1 — Supplemental table. Test of Hardy-Weinberg equilibrium for each SNP genotyped in the control and T2D study subjects. [file 1471-2350-10-33-S1.doc]

**Supplementary Table**

**Test of Hardy-Weinberg equilibrium for each SNP genotyped in the control and Type 2 Diabete study subjects**

| ***SNPs*** | Control | | T2D | |
| --- | --- | --- | --- | --- |
|  | Chi-square | P Value | Chi-square | P Value |
| ***TCF7L2***  rs7903146 | 1.460 | 0.236 | 5.650 | 0.017 |
| ***KCNJ11***  rs5219 | 0.332 | 0.665 | 0.012 | 0.935 |
| ***GCK***  rs1799884 | 0.772 | 0.408 | 0.997 | 0.346 |
| ***HHEX***  rs7923837 | 0.231 | 0.730 | 0.616 | 0.452 |
| ***ENPP1***  rs1044498 | 3.318 | 0.071 | 8.547 | 0.004 |
